# The role of the gut microbiome during host ageing

**DOI:** 10.12688/f1000research.15121.1

**Published:** 2018-07-16

**Authors:** Jens Seidel, Dario Riccardo Valenzano

**Affiliations:** 1 Max Planck Institute for Biology of Ageing, Cologne, Germany; 2 CECAD, University of Cologne, Cologne, Germany

**Keywords:** microbiome, ageing, microbiota, longevity

## Abstract

Gut microbial communities participate in key aspects of host biology, including development, nutrient absorption, immunity and disease. During host ageing, intestinal microbes undergo dramatic changes in composition and function and can shift from commensal to pathogenic. However, whether they play a causal role in host ageing and life span has remained an open question for a long time. Recent work in model organisms has revealed for the first time that gut microbes can modulate ageing, opening new questions and opportunities to uncover novel ageing-modulating mechanisms and to design anti-ageing interventions by targeting the gut microbiota.

## Introduction

**Figure 1.**
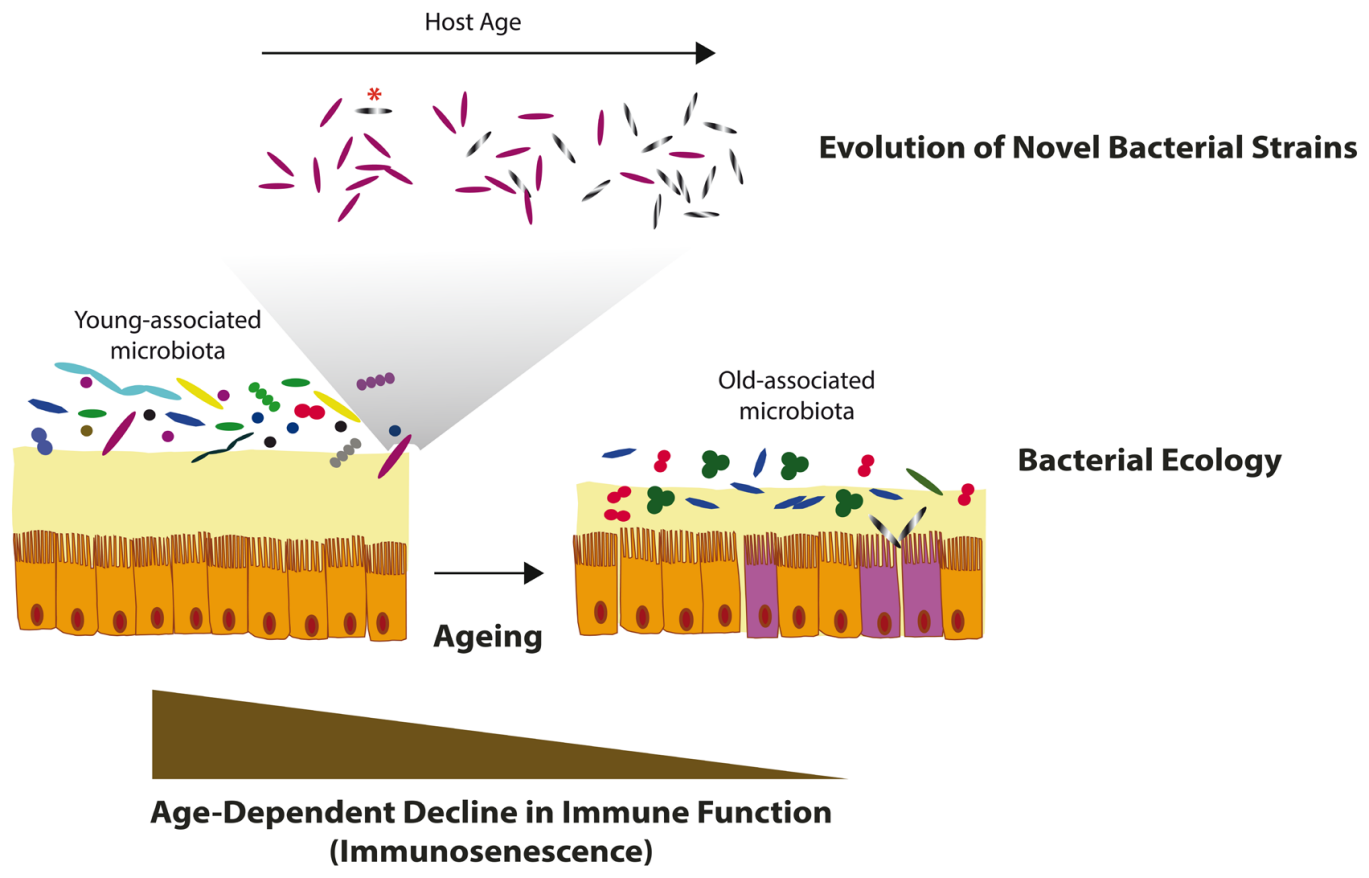
Age-dependent changes in gut microbiota. Evolutionary and ecological community changes during host ageing may play fundamental roles in shaping age-specific microbial communities. ( **Above**) *De novo* mutations (asterisk) or horizontal gene transfer in young-associated commensal bacterial species may lead to the evolution of highly fit bacterial strains that become more abundant in aged individuals, eventually leading to age-related pathogenicity. ( **Middle**) Species-species bacterial ecological interactions could affect community dynamics that shape microbiota composition throughout host life span, ultimately affecting host physiology during ageing. ( **Bottom**) An age-dependent decline in immune function may cause decreased surveillance over microbial communities over time, leading to age-dependent dysbiosis. On the other hand, a healthy microbiota itself could be necessary to preserve a healthy immune function during ageing.

In hundreds of millions of years of co-evolution, commensal microbial communities and their hosts have adapted to one another, becoming strictly inter-dependent. A sophisticated metabolic cross-talk between microbes and their multi-cellular hosts ensures balanced homeostasis and finely regulates most physiological processes. During ageing, the complex interaction between host and its associated microbial communities—termed “microbiota”—undergoes important changes, which can result in dramatic phenotypic consequences for the host, including dysbiosis, infections and overall functional decline. However, what the triggers are of this age-related host-microbiota imbalance is still poorly understood. While a young and healthy immune system is capable of efficiently maintaining a taxonomically diverse commensal microbiota, age-related immune dysfunction—that is, immunosenescence ^1^—could reduce the selection on commensal and pathogenic microbial taxa, allowing proliferation of pathobiont and pathogenic bacteria. On the other hand, ecological (species-species competitive interactions) and evolutionary (emergence of novel strains within a species) dynamics in the microbial communities of the gut could also trigger age-dependent host demise. Rather than being mutually exclusive, a combination of immunosenescence and microbiota-intrinsic population and evolutionary dynamics could lead to irreversible host dysfunctions, resulting in rapid deterioration of health and increased risk for age-related pathologies and ultimately death ( Figure 1).

## Ageing and the microbiota

Biological ageing is a multi-factorial phenomenon, consisting of the loss of homeostasis at multiple scales of biological complexity, from the molecular (for example, DNA and proteins) to the organelle, cell, tissue, organ and metabolic/system level. Both genetic and environmental factors determine ageing progression in different species. Research in laboratory model organisms has demonstrated that single gene mutations (for example, in genes in the insulin–insulin-like growth factor 1 [insulin- *IGF1*], *AMPK* and *TOR* pathways) significantly affect life expectancy and ageing ^2– 5^. Additionally, important gene variants (for example, in the *FOXO3* gene) have been associated in humans with extreme longevity ^6^. On the other hand, environmental interventions, such as dietary restriction, changes in nutrient sensing, stress and changes in temperature, can also modulate life span and ageing in experimental model organisms ^7– 10^.

Dwelling at the interface between organisms and the external environment, commensal microbes participate in several processes, including nutrient absorption ^11^, synthesis of essential vitamins, drug processing, pathogenicity, organ development ^12, 13^, circadian rhythms ^14^, and immune system maturation and modulation ^15^. Among all organs, the human gut lumen harbours the largest amount and diversity of commensal microbes, whose composition and function have been importantly associated with the modulation of the insulin signalling pathway and in general with the overall metabolic state of the host ^16^. Dramatic compositional changes occur with development in the human gut microbiota during early childhood, and the community becomes richer and more stable afterwards ^17, 18^. Despite being diverse in composition across healthy individuals, the adult gut microbial composition is considered functionally stable and involved in essential processes, such as protein translation, carbon metabolism, adhesion, amino acid and vitamin synthesis ^19^. Age-related frailty is importantly associated in humans with the loss of diversity in the core microbiota groups ^20^. Transplantations of microbes from obese individuals into mice raised in germ-free conditions lead to dramatic effects in recipient mice, including higher adiposity and differences in fatty acids and amino acid metabolism ^21^. Notably, gut microbes can effectively tune host inflammatory responses and several gut microbial taxa play a powerful anti-microbial action, suggesting a potential immune role of the gut microbiota, which can help fight infections by pathogenic bacterial species. For instance, faecal material transfer from healthy donors is successfully used in the clinic to resolve acute *Clostridium difficile* infections ^22^. Probiotic diets have been associated with beneficial life span effects in a mouse study ^23^, and human centenarians and ultra-centenarians are characterised by a gut microbial composition enriched in health-associated bacteria ^24^. Studies in yeast, flies and mice humans have shown that the gut microbiota undergoes dramatic changes during the ageing process ^20, 25– 27^, raising the question of whether these changes are a consequence or a cause of ageing. Experimental work in flies showed that, upon ageing, commensal microbes can lead to dysbiosis, which is followed by loss of barrier function and ultimately host demise ^28^. Recent work in nematode worms demonstrated that feeding worms with different bacterial species and with *Escherichia coli* mutant strains significantly tunes host life span ^29, 30^. Remarkably, despite the extensive influence of commensal microbes on host biology, very little is known about whether and how the complex microbial communities associated with vertebrate intestines affect ageing and whether they can be used to modulate ageing and life span.

A recent study conducted in a naturally short-lived vertebrate, the turquoise killifish ( *Nothobranchius furzeri*) ^31, 32^, showed that heterochronic gut microbiota transfer from young subjects to middle-aged individuals led to life span extension and delayed motor decline ^33^. Turquoise killifish undergo a wide range of age-related transformations that resemble human ageing-related phenotypes, including cancer, loss of pigmentation, reduced fecundity, neurodegeneration, cognitive decline, and cellular senescence, offering a unique opportunity to study ageing in a short-lived vertebrate ^32, 34^. Notably, unlike invertebrate model organisms, such as worms and flies, captive killifish have a very complex gut microbial community, consisting of hundreds of bacterial taxa, similar in complexity to other vertebrates, including mammals ^33^. Although young-derived gut microbiota can extend life span and delay ageing in this short-lived vertebrate model, several important questions still need to be answered. Is the life span modulatory role of the gut microbes amplified in short-lived species or is this a more general mechanism that applies broadly to other vertebrates, including mammals? Do gut microbes modulate host ageing in vertebrates via conserved ageing pathways or via novel mechanisms?

## Possible mechanisms by which the gut microbiota can modulate host ageing

Pioneering work in nematode worms showed that mitochondrial unfolded protein response is the target of a key microbial metabolite, colanic acid, whose production leads to extended worm longevity ^29^. Mice raised on a life-long dietary restriction regime—typically associated with longer life span—have a significantly altered gut microbiota, characterised by lower abundance of bacterial taxa negatively associated with life span and a higher representation of bacteria of the genus *Lactobacillus* ^35, 36^. Transfer of germ-free mice with conventional specific pathogen-free microbiota induces high levels of serum IGF1, suggesting a direct connection between gut microbiota and the metabolic activation of canonical ageing pathways ^37^. Similarly, high levels of health-beneficial short-chain fatty acids lead to serum upregulation of IGF1, further supporting a strong mechanistic link between the insulin- *IGF1* pathway and gut microbial metabolism ^37^. Short-chain fatty acids generated by commensal gut microbes induce anti-inflammatory responses ^38^, protecting from bacterial and fungal infections ^39^ and leading to life span extension in worms ^40^. Through similar mechanisms, young-associated gut microbes may induce a healthier state and a slower ageing rate ^41^. The health-span–promoting drug rapamycin also has anti-inflammatory actions ^42^ and, when transiently administered to middle-life mice, significantly reshapes the gut microbiota, leading to increased abundance of segmented filamentous bacteria in the small intestine ^43^. The gut microbiota could in fact affect ageing and life span via its action on the immune system, modulating pro- and anti-inflammatory responses, importantly associated with host ageing ^44^. Studies in gnotobiotic mice have helped elucidate the contribution of different components of complex gut microbiota in modulating host’s metabolism and physiology (for instance, in the case of inflammatory bowel disease–induced dysbiosis) ^45^. In gnotobiotic mice, single microbial taxa (for example, *Bacteroides thetaiotamicron* and *Faecalibacterium prausnitzii*) play complementary roles in the gut and can lead to specific metabolic alterations in gut epithelial mucus production and in short-chain fatty acid synthesis and consumption, which could be importantly linked with the risk for ageing-related pathologies ^46^. Colonising the gastrointestinal tract of laboratory mice with microbiota from wild mice, investigators were able to reduce inflammation, promoting host fitness and survival after lethal viral infections and against colitis-associated tumorigenesis ^47^. These results raise the question of whether similar effects could be induced by maintaining a highly diverse, young-associated intestinal microbiota throughout mice ageing. A functioning inflammasome and B-cell compartment are key to shaping the gut microbiota composition, as shown in experiments conducted in mice lacking *Nlrp6* and *RAG2*, respectively ^45^. Specifically, inflammasome and adaptive immune function were essential to shaping the microbiota in the presence of pro-inflammatory bacteria ^45^. It is possible that, during ageing, immune function shifts towards inflammatory responses against commensal bacteria, leading to host-microbiota disbalance. Similarly, chronic inflammation is associated with a higher risk for age-associated diseases ^44^. In the context of infection, immune tolerance for commensal bacteria indeed shifts towards inflammation, compromising this delicate host-microbiota balance ^48^. Systemic translocation of the gut pathobiont *Enterococcus gallinarum* in a mouse model predisposed to autoimmunity has been causally associated with triggering of autoimmune responses, further providing a mechanistic connection between microbiota composition and host immune status ^49^.

Immunosenescence may indeed lead to a failure in maintaining commensal microbiota structure and function; on the other hand, microbial-intrinsic population dynamics—which could lead to the emergence of more pathogenic bacteria—may trigger immune failure and eventually induce functional decline in the host. Longitudinal studies in human samples over a six-month period have shown that gut microbial communities are rather stable, and within-host bacterial evolution is the consequence of horizontal gene transfer among bacterial strains from the same microbiota rather than occurring from *de novo* mutations or introgression from bacteria resident in other hosts ^50^. If this approach is extended to the study of bacteria throughout host life span, it could be possible to ask whether microbes evolve during host ageing and whether higher virulence—associated with pathogenicity in older subjects—is the consequence of the microbial evolution to evade immune surveillance.

Overall, the study of the host-microbiota dynamics throughout ageing can help reveal novel physiological and molecular mechanisms that contribute to the maintenance of homeostasis and ultimately help design powerful and personalised interventions that target the microbiota as a novel fundamental player implicated in the regulation of host ageing processes.

## Open questions

The intimate connection between host physiology and commensal microbial function supports the implication of host-associated microbiota in the majority—if not the entirety—of biological processes of the host, including ageing. The study of the microbiota in the context of host ageing is a novel field of research and sets itself at the interface of several fields of investigation, including medical microbiology, immunology, ecology, evolutionary and population genetics, tissue and cell biology, physiology, nutrition, and metabolic research. Several confounders affect the microbiota changes occurring during human ageing, including age-dependent dietary changes, drug use, changes in mobility and housing conditions (community dwelling or elderly care facilities). Beyond descriptive connections between microbial composition and host health status, very few studies to date have dissected the causal role of the gut microbiota during ageing. To unweave the complex functional connection between host and microbiota in the context of ageing, it will be of paramount importance to study the role of not only bacteria but also archaea, viruses, fungi and microbial eukaryotes living between complex multi-cellular hosts and their environment. This holistic understanding of community dynamics can help reveal the intricate ecology of health, disease and ageing processes. To this end, it will be key to adopt novel experimental and analytical approaches to study the impact of different complex microbial communities on the host. Ultimately, by acting on microbial composition, nutrition and the immune system, it will be possible to test the efficacy of novel interventions to beneficially impact ageing and delay the onset of age-related pathologies.
